# Advancements in Agricultural Ground Robots for Specialty Crops: An Overview of Innovations, Challenges, and Prospects

**DOI:** 10.3390/plants13233372

**Published:** 2024-11-30

**Authors:** Marcelo Rodrigues Barbosa Júnior, Regimar Garcia dos Santos, Lucas de Azevedo Sales, Luan Pereira de Oliveira

**Affiliations:** Department of Horticulture, University of Georgia, Tifton, GA 31793, USA; regimar.garcia@uga.edu (R.G.d.S.); lucas.sales@uga.edu (L.d.A.S.); luan@uga.edu (L.P.d.O.)

**Keywords:** ground robots, fruits, vegetables, horticulture, literature review

## Abstract

Robotic technologies are affording opportunities to revolutionize the production of specialty crops (fruits, vegetables, tree nuts, and horticulture). They offer the potential to automate tasks and save inputs such as labor, fertilizer, and pesticides. Specialty crops are well known for their high economic value and nutritional benefits, making their production particularly impactful. While previous review papers have discussed the evolution of agricultural robots in a general agricultural context, this review uniquely focuses on their application to specialty crops, a rapidly expanding area. Therefore, we aimed to develop a state-of-the-art review to scientifically contribute to the understanding of the following: (i) the primary areas of robots’ application for specialty crops; (ii) the specific benefits they offer; (iii) their current limitations; and (iv) opportunities for future investigation. We formulated a comprehensive search strategy, leveraging Scopus^®^ and Web of Science™ as databases and selecting “robot” and “specialty crops” as the main keywords. To follow a critical screening process, only peer-reviewed research papers were considered, resulting in the inclusion of 907 papers covering the period from 1988 to 2024. Each paper was thoroughly evaluated based on its title, abstract, keywords, methods, conclusions, and declarations. Our analysis revealed that interest in agricultural robots for specialty crops has significantly increased over the past decade, mainly driven by technological advancements in computer vision and recognition systems. Harvesting robots have arisen as the primary focus. Robots for spraying, pruning, weed control, pollination, transplanting, and fertilizing are emerging subjects to be addressed in further research and development (R&D) strategies. Ultimately, our findings serve to reveal the dynamics of agricultural robots in the world of specialty crops while supporting suitable practices for more sustainable and resilient agriculture, indicating a new era of innovation and efficiency in agriculture.

## 1. Introduction

Specialty crops include a wide variety of fruits, vegetables, tree nuts, and horticulture and nursery crops, including medicinal plants and floriculture crops [1]. These crops span a large number of commodities, markets, and supply chains [2]. Notably, this biodiversity plays an important role in food security [3]. Differing from row crops, specialty crops have unique characteristics, nutritional values, and market demands, which make their cultivation a particular and costly procedure [4]. Specialty crop farms experience the highest labor costs, accounting for nearly 40% of their total cash expenses, which is approximately three times more than the costs for field crops [5]. Furthermore, some specialty crops present specific management needs, for example, trellising and pruning, which increase the care and cost of production, adding more challenges to their cultivation. Yet an intriguing factor is the ability of specialty crops to produce high-value products in relatively small areas [6]. However, it is noteworthy that the lack of machinery to support specialty crop cultivation poses one of the most crucial challenges, particularly those based on autonomous operations.

Essential agricultural tasks for specialty crops, such as planting, pruning, spraying, and harvesting, are still performed manually. Such tasks make the production of these crops labor-intensive and expensive. Certainly, the challenges tend to increase when field activities are performed under adverse weather conditions such as extreme temperatures (heatwaves and freezing conditions). Those conditions can considerably reduce labor safety and increase negative personal impacts [7]. Consequently, there is an urgent need for innovative technological solutions in specialty crop fields. By reviewing the scientific literature on specialty crops, we could identify a portion of up-and-coming topics to support their cultivation, especially the integration of robots as an emerging and promising technological advance [4,8].

Agricultural robots, also referred to as agribots or unmanned ground vehicles (UGVs) [9,10], represent cutting-edge technology for performing a wide range of agricultural tasks. These machines are generally equipped with a variety of components, traditionally organized into four main categories: vision systems, control systems, mechanical actuators, and mobile platforms [11,12]. These components play an important role in making a robot functional and able to perform an activity. However, with the advancements in autonomous robotics, these systems are now integrated with more sophisticated technologies, mainly including a wide range of different cameras and sensors (e.g., RGB, multispectral, hyperspectral, LiDAR, etc.) to facilitate plant monitoring [13]. In short, these machines are emerging as a timely technology for improving efficiency, precision, and sustainability, contributing positively to environmental [14] and social impacts [15]. Agricultural robots have the potential to improve specialty crop production by providing modern solutions for tasks such as harvesting [16], pruning [17], weeding [18], spraying [19], and fertilizing [20]. Additionally, they are adaptable and customizable to meet the specific needs of different crops and environments, and they are capable of operating both partially and fully autonomously [21]. This autonomy simplifies agricultural operations and alleviates labor shortages while reducing labor costs for farmers. Moreover, autonomous operation allows robots to work around the clock, maximizing productivity and throughput. This adaptability enables better performance in agricultural operations, allowing producers to dynamically respond to changing conditions and demands. Furthermore, combining these robots with advanced sensing systems and positioning technologies ensures that they achieve high levels of precision in their tasks, a milestone expected in modern agriculture.

Numerous studies have explored agricultural robots in specialty crops. This proves that the benefits of this technology are evident. Additionally, some review papers have stated that the robots contribute to weeding [22] and harvesting [8] and described their social and ethical impacts [15]. However, to the best of our knowledge, an overview synthesizing the state of the art of agricultural robots for specialty crops remains absent from the literature. Furthermore, there is a notable gap in existing reviews that focus on the primary areas of application, the specific benefits they offer, the challenges they encounter, and the extent of global collaborative efforts in this field. Therefore, we aim to fill this gap by providing a wider overview of the applications of agricultural robots for specialty crops. This review offers insights into the latest advancements, challenges, and opportunities within each agricultural operation.

This paper addresses the following questions:

i. What is the current state of research on agricultural robots tailored to specialty crops in the literature?
ii. Which journals are most active in publishing research on agricultural robots for specialty crops?
iii. Which countries are the primary contributors to scientific publications on agricultural robots for specialty crops?
iv. Which specific crops derive the most benefits from agricultural robots?
v. What are the primary tasks for which agricultural robots are increasingly being deployed in specialty crop production?
vi. What are the benefits, limitations, and future research opportunities in the field of agricultural robots for specialty crops?

So far, we have highlighted the importance of specialty crops and how agricultural robots can potentially contribute to their production, emphasizing the need for a comprehensive state-of-the-art study on this topic (Section 1). In the following sections, we describe in detail the methodology and selection criteria employed to ensure a systematic and thorough analysis of the literature (Section 2). This is followed by a critical analysis of our findings, focusing on the progress of literature, the most productive journals, global scientific collaboration, the main target crops, the most popular subject, and the commercial availability of robots for specialty crops (Section 3). Finally, we present a robust discussion that summarizes the key discoveries, highlights the advantages and limitations of agricultural robots for specialty crops, identifies opportunities for future research, and concludes with the implications of our findings for advancing the field (Section 4).

## 2. Methodology

### 2.1. Literature Guidelines

**Figure 1:**
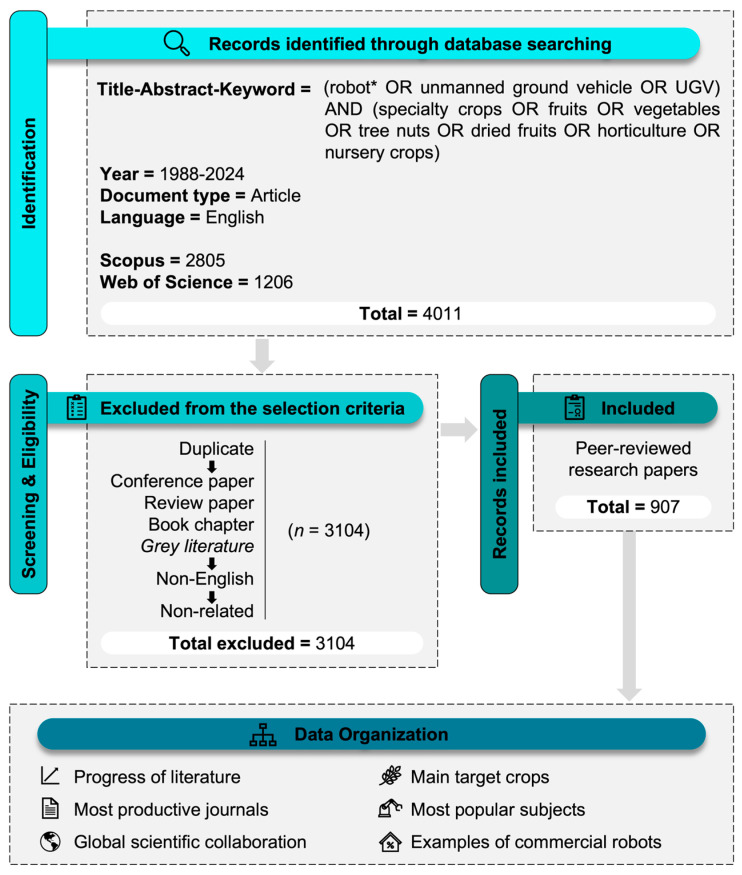
Flow diagram of studies’ screening and selection.

Our review was conducted through an in-depth examination of the scientific literature on agricultural robots and specialty crops, following the Preferred Reporting Items for Systematic Reviews and Meta-Analyses (PRISMA) [23] guidelines (Figure 1).

### 2.2. Search Strategy

The databases Scopus^®^ and Web of Science™ were selected for the retrieval of scholarly items. A representative search strategy was constructed by combining indexing terms and Boolean operators. The search string was as follows: [title-abstract-keyword = (robot* OR unmanned ground vehicle OR UGV) AND (specialty crops OR fruits OR vegetables OR tree nuts OR dried fruits OR horticulture OR nursery crops)]. The selected indexing terms are comprehensive, and they ensured the representativeness of the relevant literature. It should be noted, however, that some relevant papers may have been excluded due to the specific emphasis on the term “robot”. Nevertheless, this level of specificity is consistent with the primary objective of our review, which is centered on the applications of ground robots. Furthermore, the review encompasses the period between 1988 and 2024. As the year 2024 is still in progress, the review includes all papers published up to 21 October 2024. This approach ensured the inclusion of a comprehensive bibliographic collection, encompassing literature from the advent of robots in the field of specialty crops to the most recent publications.

### 2.3. Selection Criteria

The initial search identified 4011 potential studies. We exported the database into Microsoft Excel and organized the spreadsheet by topic, including author, title, year of publication, journal, and DOI. Initially, we excluded duplicates to prevent bias. Subsequently, our reviewers (M.R.B.J., R.G.d.S., and L.d.A.S.) conducted an independent assessment of the studies for consistency and eligibility. Therefore, they excluded conference papers, review papers, and book chapters to ensure that only peer-reviewed research papers were included, as these typically undergo a more rigorous review process. Additionally, non-English language papers and any “grey literature” (e.g., reports, theses, and non-peer-reviewed articles) were excluded to maintain the quality and reliability of our review. For the remaining studies, we conducted a full-text review to ensure that they fit within our scope, which included research on agricultural robots for specialty crops. Consequently, studies that did not focus on robot application or were unrelated to specialty crops were excluded. As a result, 3104 studies were removed, and 907 were deemed eligible.

### 2.4. Database Organization and Discussion

The 907 eligible studies were saved in a list on the SciVal platform (https://www.scival.com/ (accessed on 30 October 2024)) to perform a bibliometric analysis according to the following criteria: year of publication, journal, country, crops, and subject. Additionally, to enhance the depth of our analysis, we also obtained qualitative data via SciVal, including the number of citations, the field-weighted citation impact (FWCI) associated with the studies, and performance indicators, namely outputs in the top 10 citation percentiles, publications in the top 10 journal percentiles, and geographical collaboration. Particularly, by tracking journal citations, we can identify the most influential studies and journals in the field, providing insights into the main sources of knowledge and trends in a specific topic. Furthermore, the analysis of geographical collaboration allows us to map the network of institutions worldwide, highlighting significant collaborations that drive innovation in this area. Finally, the results are presented in independent sections.

### 2.5. Data Availability

Our database is organized by topics and is available to independent researchers and readers (https://doi.org/10.17632/482j99rfpz.4).

## 3. Results

### 3.1. Progress of Literature

**Figure 2:**
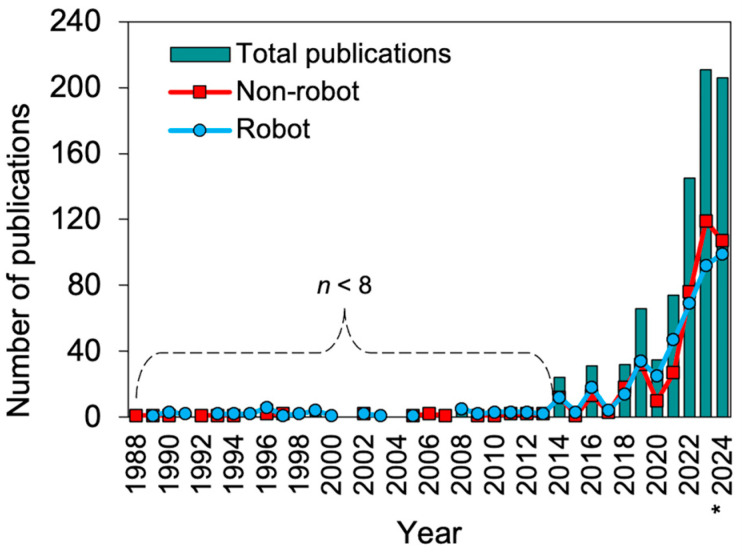
Number of publications on robots for specialty crops from 1988 to 2024. Publications were classified as studies that used robots (robot) and those that only supported robot applications (non-robot). * The publications for the year 2024 cover the period from January to 21 October 2024.

All 907 papers meeting the eligibility criteria were subjected to a comprehensive categorization based on the year of publication (Figure 2). The application of agricultural robots for specialty crops has experienced a period of fast evolution. The results demonstrated a consistent trend, regardless of whether the research involved the use of a robot (robot) or the development of a system that supports robot applications (non-robot). In the initial period between 1988 and 2013, there was a relatively low level of interest in this agricultural technology, with a total of less than eight studies published. However, it is evident that progress has occurred over the past decade, particularly with the increasing peaks observed in 2022 and 2024.

A noteworthy deviation from the established trend was observed in 2020, which can be attributed to the significant impact of the global pandemic caused by the virus that causes COVID-19 [4,24,25]. As a result of protective measures and a prolonged period of strict lockdowns, universities and research centers were closed. Furthermore, export and import restrictions were significantly tightened, impeding the transfer of products and devices. This global crisis had a profound impact on the continuity of ongoing projects and the development of new research worldwide.

From the 907 eligible papers, 465 (51.27%) directly applied or developed robots for specialty crop applications. Certainly, these numbers emphasize the progress of robots toward practical application. Conversely, 442 papers (48.73%) did not directly use robots for specialty crops. However, they support the application of robots, for instance, through the development of vision systems. Moreover, these 907 papers contributed to 19,612 citations, and 42.2% of the papers were in the top 10% of the most-cited publications. Furthermore, a remarkable FWCI of 2.97, ranking 197% above the global average research performance, highlights the scientific community’s interest in the subject matter.

### 3.2. Most Productive Journals

**Figure 3:**
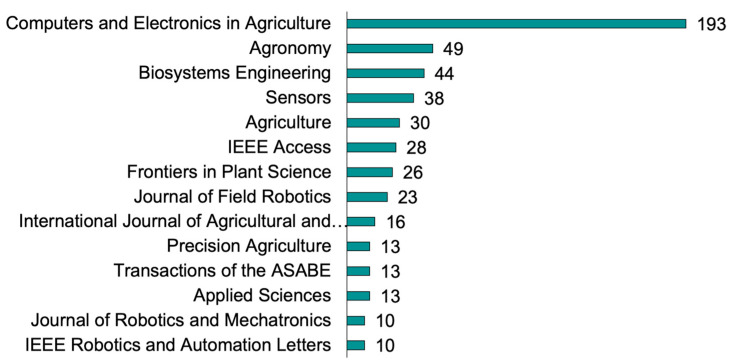
Number of publications by journals on robots for specialty crops from 1988 to 2024. Journals that contributed 10 or more publications are highlighted in the graph.

A total of 214 journals published research related to agricultural robots for specialty crops. However, a select group of 14 journals collectively accounted for 56% of the total papers (Figure 3). Remarkably, *Computers and Electronics in Agriculture* (Elsevier) achieved first place with the highest number of papers (n = 193). This result is likely attributed to the journal’s relevance and scope; it largely covers and supports robotic applications. The second most productive journal was *Agronomy* (MDPI), with 49 papers, closely followed by the following: *Biosystems Engineering* (Elsevier), with 44 papers; *Sensors* (MDPI), with 38 papers; and *Agriculture* (MDPI), with 30 papers. The remaining journals produced fewer than 30 papers. It is noteworthy that 42.6% of the papers were in the top 10% of the most-cited journals.

### 3.3. Global Scientific Collaboration

**Figure 4:**
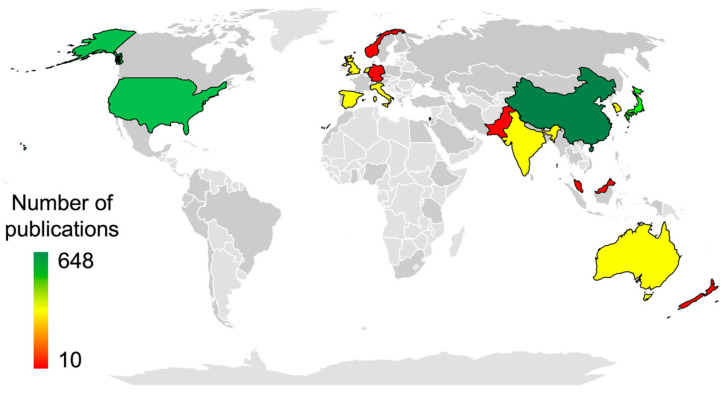
Number of publications worldwide on robots for specialty crops from 1988 to 2024. Countries contributing 10 or more publications are highlighted with colors from red to green. Countries with fewer than 10 publications are shown in dark gray, and countries that did not produce publications are shown in light gray.

In a comprehensive analysis of global scientific collaboration in the 907 studies (Figure 4), all continents demonstrated active participation, reflecting a global engagement and dissemination of agricultural robotics for specialty crop production. A total of 71 countries contributed to this field of study. China emerged as an outstanding contributor, representing 71% of the total publications (*n* = 648). Subsequently, the United States ranked second with 103 studies. Notable contributions were also observed from Japan (62), Australia (31), India (29), United Kingdom (28), and Spain (27). Additionally, Israel, South Korea, and the Netherlands contributed equally to 21 papers. Other countries contributed 20 or fewer papers individually. In terms of geographical collaborations (authorship), 17.6% of the papers involved international collaboration, 45.1% only national collaboration, 35.7% only institutional collaboration, and 1.6% no collaboration (single authorship). Additionally, only 1.4% of the papers involved collaboration between academic and corporative affiliations.

### 3.4. Main Target Crops

**Figure 5:**
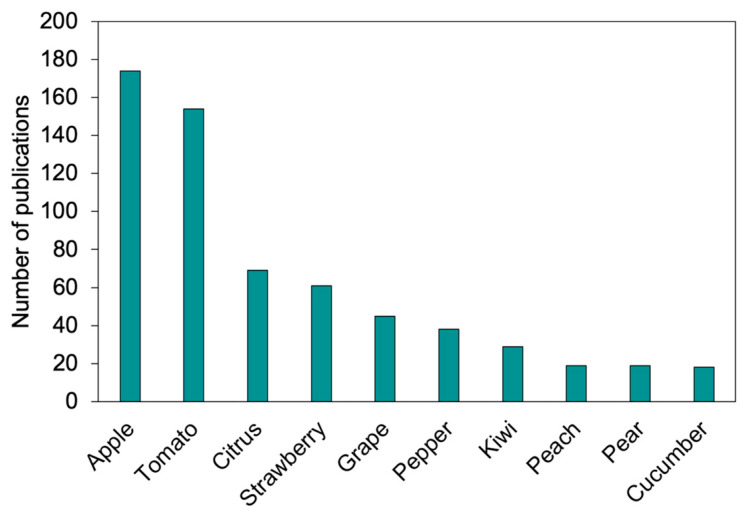
Number of publications on robots for specialty crops from 1988 to 2024 specifying the top 10 crops.

Approximately 80% of the investigated papers on agricultural robots for specialty crops proposed specific developments related to particular crops, rather than general applications. However, some specific crops have attracted more attention from researchers (Figure 5). Apple garnered the most attention, with 174 papers dedicated to the implementation of agricultural robots in its production. Following closely behind was tomato, featured in 154 papers. In third place, although at a more distant margin, citrus was the focus of 69 papers. Subsequently, strawberry was explored in 61 papers, followed by grape in 45 papers, pepper in 38 papers, and kiwi in 29 papers. Other crop varieties received comparatively less attention, with individual contributions of fewer than 20 papers.

### 3.5. Most Popular Subjects

**Figure 6:**
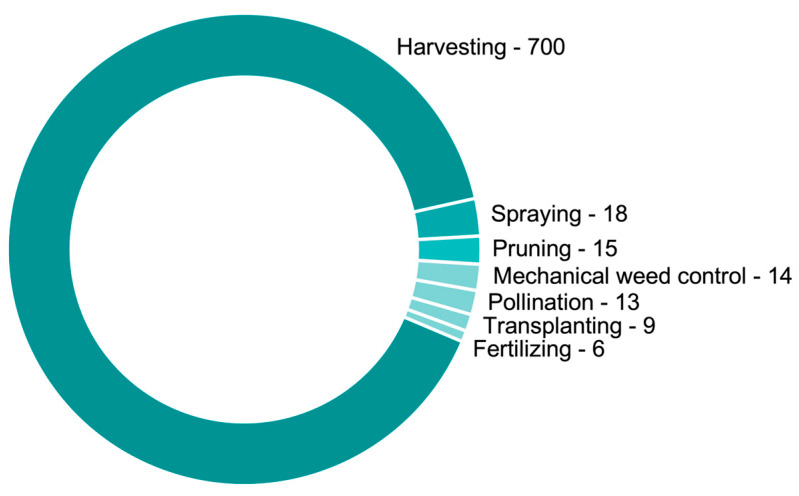
Number of publications on robots for specialty crops from 1988 to 2024 specifying subjects.

It is noteworthy that the primary focus of research in the domain of agricultural robots for specialty crops was predominantly for the harvesting operation (Figure 6). Approximately 80% of the investigated papers either utilized harvest robots or addressed applications related to their support, representing an impressive scientific output of over 907 papers. Conversely, other subjects individually accounted for less than 2% of the overall research interest. For instance, spraying robots emerged as the second most prominent area of interest, with 18 papers dedicated to the topic. Additionally, similar scientific contributions were found for pruning (15), mechanical weed control (14), and pollination (13). Furthermore, timely opportunities were identified in robot applications such as transplanting (9) and fertilizing (6). To further understand the progress in each of these areas, we elaborated a discussion highlighting the advancements and strengths (Section 3.5.1, Section 3.5.2, Section 3.5.3, Section 3.5.4, Section 3.5.5, Section 3.5.6 and Section 3.5.7). Weed control, in particular, is a broader topic that benefits not only specialty crops. We recognize that robotic technologies focused on weed control share similar advances for specialty or row crops (e.g., spot-spraying, laser weeding, and mechanical weeding). However, the 14 papers identified in our search developed relevant and unique mentions for specialty crops. Consequently, they are mentioned throughout this literature review.

#### 3.5.1. Harvest

In recent years, there has been a growing interest among researchers in developing harvesting robots for specialty crops. However, scientific efforts were conducted over three decades ago on this topic [26,27,28]; a real-time fruit-tracking system was developed to estimate the size and position of a fruit region based on color images. Despite this early groundwork, challenges persist for harvesting robots, particularly when applied to specialty crops, given their inherent variability across different crop types. For instance, the harvesting process for herbaceous crops involves cutting and separating the useful part from the rest, whereas, for tree crops, such as fruits, delicate and smooth separation from the tree is required without compromising the structural integrity of the tree and the fruit, thereby ensuring productive continuity and post-harvest quality, respectively. This complexity is intensified due to various agronomic characteristics common to tree crops [29].

The development of mechanized and mechanical systems for specialty crop harvesting faces challenges, particularly for fruit picking. Several factors, such as low work efficiency, low success rates, fruit damage, challenges in detection under unstable illumination, and high costs, make this task a concern, as indicated in previous studies on robotic harvesting for strawberries [30]. Additionally, the challenges tend to intensify when methods for fruit recognition are added. For instance, previous machine vision research focused on estimating the three-dimensional (3D) pose of free-form objects [31]. Despite their innovative approach, the authors [31] faced further challenges for fruits due to significant shape variations and discrepancies from the model. Therefore, future research would be necessary to refine or even develop a novel 3D method for automated harvesting.

Given the challenges posed by fruits’ similarity in color to branches and leaves, as well as their susceptibility to shading and overlapping, the accurate detection and localization of fruits present significant difficulties. To address this issue, a convolutional neural network (CNN) algorithm (Mask R-CNN) was employed for the detection and segmentation of mature green tomatoes [32]. This approach involves the deployment of a mobile robot designed to capture images around the clock under varying conditions within a greenhouse, thereby ensuring the diversity of the dataset. More recent advancements introduced autonomous rover systems capable of detecting and estimating fruit ripeness in natural crop fields through visual information [33]. This technological innovation supports the identification of the current fruit maturity and facilitates the selection of the best fruits for harvesting, minimizing damage in the process [34]. Additionally, efforts have been made to support the harvesting of soft fruits [35]. A similar approach proposed a you only look once (YOLO)-based blueberry ripeness detection algorithm suitable for integration into a blueberry-picking robot [36]. Their algorithm demonstrated good transferability and applicability, suggesting its potential for use with other fruits as well. However, a major challenge in the application of harvesting robots is their performance under real-world conditions. Therefore, some efforts have been made to improve the performance of these robots through lightweight algorithms. This approach allows for better performance of the robots under different light conditions, different fruit shapes, and occlusion due to leaves [37,38,39]. Additionally, this approach requires relatively low computational demands.

#### 3.5.2. Spraying

Spraying operation is a critical aspect to be considered during specialty crop production, a timely task to potentially promote plant health by controlling pests or disease infestations. Numerous spraying devices and tools are commercially available; however, most of them still exhibit limitations regarding their effectiveness and adaptability for specific crops. Traditional spraying methods, particularly present in developing countries or small fields, often involve backpack sprayers, which increase chemical exposure for workers and tend to be labor-intensive, as well as difficult when controlling the optimum amount of product applied. Alternatively, heavy machinery, such as self-propelled or tractor-mounted sprayers, are employed for spraying tasks; however, this potentially increases soil compaction and crop trampling. Furthermore, the financial implications of acquiring large machinery must also be considered. In response to these challenges, spraying robots emerge as a promising solution. These autonomous systems significantly enhance spraying operations in specialty crop production while mitigating the drawbacks associated with traditional methods.

Initial studies on spraying robots explored the use of modular agricultural robot systems [40]. These systems were equipped with end-effectors and image-based sensors (red, green, and near-infrared) for disease detection and subsequent spraying, making them precision spraying systems. Their investigation showed timely results, validating the hypothesis that such robots can automatically detect and spray desired areas within a canopy. As a result, they significantly reduce pesticide usage compared to conventional broadcast-spraying methods. Furthermore, a recent study aimed to develop a new control algorithm for a robotic rover, also an image-based recognition system for localized spraying tasks [41]. A notable advantage of their study was lower-resolution image processing, which considerably decreased the cost of expensive high-resolution cameras and hardware requirements for processing.

Another timely approach employed a single 3D light detection and ranging (LiDAR) sensor to detect fruit tree information [42]. The precision variable-rate spraying (PVS) system reduced pesticide application volumes, ground losses, air drift, and environmental pollution, with the particularity of being an automatic navigation system. Similarly, another study proposed the use of LiDAR as a single sensor in a prototype robot for an artificial tree-based orchard [43]. The application of machine learning (ML) algorithms was crucial to enabling real-time path planning. Additionally, deep learning (DL) techniques were integrated into a variable-rate control system [44]. The main purpose was to segment fruit trees in pear orchards and spray them effectively. As a result, field tests demonstrated satisfactory performance for all open, on/off control, and variable-rate control.

Following the real-time solutions, another approach developed a crop signaling concept utilizing machine vision for targeted application through a precision micro-jet sprayer [45]. The trial was performed indoors, and the results achieved a rate of 98% of the targets being properly sprayed. A novel method based on multi-object tracking and segmentation (MOTS) was proposed for segmenting and tracking multiple vegetable plants [19]. This method incorporated contour and blob features to re-identify objects, ensuring that each vegetable was sprayed only once during robot travel. While initially tested on lettuce farms, the method holds promise for application to similar leafy greens. Similarly, a YOLO model was developed and integrated into a robot for real-time strawberry fruit detection and simultaneous pesticide spraying [46]. The mobile robot was developed for a greenhouse environment, and the results showed great performance, reaching accuracies above 97%.

#### 3.5.3. Pruning

Pruning is a critical task in the cultivation of most fruit and tree nut crops. This practice is essential for enhancing yield and fruit quality by eliminating the excess of low-productive branches. Improved air circulation and sunlight penetration within the canopy resulting from pruning can potentially enhance fruit color, size, and flavor while contributing to reducing disease incidence. Furthermore, pruning plays a vital role in disease and pest management by facilitating the removal of infected branches and improving spray penetration. Despite its importance, traditional pruning operations are typically performed manually, presenting challenges such as labor intensity, high costs, and time consumption. Consequently, the development of pruning robots has emerged as a promising solution to assist specialty crop growers, particularly fruit and nut tree growers, in addressing these challenges for optimizing pruning practices. By automating this labor-intensive task, pruning robots have the potential to enhance efficiency, reduce costs, and improve overall productivity in fruit crop cultivation.

As an initial concern, a comprehensive analysis was developed to understand the relationship between agronomic characteristics, working environments, transportation logistics, residual branch treatment, and the weight of the end effector of the pruner [47]. The results proved effectiveness during the pruning task, as well as the ability to keep the surfaces of the branches completely smooth, which allows natural restoration. Additionally, the robot also demonstrated accuracy when crushing the pruned branches.

Common challenges faced by agricultural robots’ applications are the irregular sizes, shapes, and growing conditions of trees. In response, recent efforts considered this concern in the context of pruning and developed an innovative approach to achieving customizable pruning patterns in traditional orchards [48]. One timely approach implemented a robot with a hydraulic disk-saw. Additionally, the authors implemented a continuous prediction algorithm to precisely control the movement of the hydraulic cylinders, consequently enabling pruning to meet the specific requirements of the orchard. Another approach was developed through a vision system capable of generating dimensionally accurate 3D models of plants sufficient for measuring key pruning metrics, particularly for stem pruning [17]. The results demonstrated the potential of the robotic platform to generate accurate plant metrics for future decision-making and automatic pruning action. Similarly, a semi-autonomous system was proposed for pruning modern planar orchard architectures using simple pruning rules [49]. This system used a new segmentation method to extract the branches from the foreground of the scene, without the need to control the lighting of the environment. The results demonstrated efficacy in detecting and pruning targets with minimal environmental control. Most recently, a method for automated pruning strategies was developed for cherry trees [50]. This approach integrated a space colonization algorithm, which enabled the precision segmentation of the tree. Compared to other pruning methods, this study showed superior results, achieving an overall accuracy of 85%.

#### 3.5.4. Mechanical Weed Control

Weed control is a crucial task with the primary objective of mitigating competition for water, nutrients, and light. Traditionally, spraying is a prevalent method for weed management in specialty crops; despite its effectiveness, it contributes to plant contamination and also does not support organic cultivation. Conversely, manual labor or mechanical tools have been a usual method of weed control in organic operations, such an arduous and time-spending task. Additionally, some mechanical methods, such as mechanical fingers or hoes, have been greatly used; however, they have the potential to cause damage to these crops. Given the inherent fragility of specialty crops, weed management practices must be reshaped to provide these crops with better conditions for development. This highlights the need for alternative approaches. In this context, investing in robots for weed control presents a timely opportunity.

A notable study evaluated the effectiveness of a robot compared to a standard cultivator within a complete integrated weed management (IWM) system [51]. The results were compared in terms of crop stand after cultivation, crop yield, weed control efficacy, and hand-weeding time. The findings revealed that the robot removed 18 to 41% more weeds at moderate to high weed densities and reduced hand-weeding times by 20 to 45% compared to the standard cultivator. However, at low weed densities, only slight differences were noted between them. Furthermore, an approach integrated a novel crop signaling technique and an autonomous robot system for a mechanical weeding task [52]. This approach detected the stem emerging points (SEPs) of tomato plants in outdoor environments and achieved a success rate of 99.19%, also supporting real-time robotic weed control. Lastly, an interrow weed detection method was applied based on the YOLO algorithm [53]. The authors’ approach focused on detecting uncut weeds and posterior management using a robot. The results demonstrated effectiveness >80%.

As robots grow in popularity and demonstrate interesting performance for weed control, some concerns also emerge, especially regarding their compatibility, connectivity, data transfer, and communication. While this concern might arise beyond the context of weed control, recent research focused on this particular context through the use of the ISO 11783 standard [18]. This specific study proposed the development of middleware that would facilitate the integration of a weeder robot with a combination of a sprayer and a retrofitted tractor, a notable contribution to enhancing operational efficiency in agricultural robotics systems.

#### 3.5.5. Pollination

Ensuring the production of specialty crops, particularly fruits, depends on the meticulous process of pollination. Physiologically, pollination is the pivotal stage whereby pollen grains are transferred from the male anther of a flower to the receptive female stigma. While it is naturally facilitated by a diverse array of pollinators, including honeybees, butterflies, birds, bats, and various other animals, challenges can emerge in certain field locations or under specific weather conditions where natural pollinators can fail to efficiently execute their vital role. This presents an open challenge for specialty crop production, highlighting the critical need for innovative solutions. In response to this challenge, investing in robots presents a new opportunity for pollination solutions. By leveraging robotics technology, it becomes possible to develop specialized robots capable of assisting or even replacing natural pollinators in pollination tasks.

While the concept of robotic pollinators is still in its beginning, ongoing R&D efforts hold promise for addressing pollination challenges in specialty crop production. By embracing this innovative technology, farmers and growers can enhance the reliability and resilience of their pollination processes, ultimately ensuring the continued success and sustainability of specialty crop production. For instance, a pollinator robot capable of autonomously targeting and artificially pollinating flowers within a kiwifruit canopy was developed [54]. Their study demonstrated that this pollination system could effectively target and pollinate 79.5% of flowers.

Additionally, a pollination system based on a robotic arm was developed [55]. This system included lightweight kiwifruit detection, allowing pollination operations according to the position and orientation of the flowers. Furthermore, aiming to improve pollen utilization efficiency, a new approach was introduced using a liquid robotic pollinator [56]. Their system consisted of a pneumatic, hydraulic cylinder for the control of the pollen suspension dosage and internal-mixing air-assisted nozzles for pollen suspension spraying. Similarly, another pollinating robot was developed [57], but it included a vision system, an air–liquid spraying system, a mechanical arm, a crawler-type chassis, and a control system. This complete system could effectively perform pollination by selecting suitable flowers and precisely targeting their pistils. In addition to the field application, robots are also suitable for greenhouse applications, which is a noteworthy advance, considering such a place where natural pollinators are not effective. Therefore, a study proposed a suspended pollination robot for self-pollination in tomato plants [58]. The robot consists of a dual-camera system and an actuator. One of the cameras is used to calibrate the manipulator’s position toward the flowers, while the second one is used to recognize the position and orientation of the flowers. The results showed a 92% success rate.

#### 3.5.6. Transplanting

Transplanting is an essential and required process for many specialty crops. It is defined as a procedure of relocating seedlings from trays or beds to the field. However, traditional transplanting methods are carried out predominantly by hand, which requires intensive field labor. Consequently, this approach is naturally time-consuming and laborious, and often fails to achieve uniformity across a field. Conversely, semi-autonomous machines have been developed to support transplanting tasks. Although helpful, they are still highly dependent on human intervention. Consequently, the emergence of robotic technology presents a promising solution for addressing these limitations.

Initial research efforts focused on developing innovative devices for automating the transplanting process [59]. This advancement involved the creation of a pincette-type pick-up prototype. However, the efforts were mainly dedicated to transferring seedlings from high-density trays to low-density growing trays or pots. The results demonstrated the high influence of the root-lump moisture content, penetration angle, seedling age, extraction speed, grasping force, and penetration depth being of lesser importance. Notable approaches introduced a robot arm tailored to handling paper-pot seedlings [60,61]. The flexible robot arm allowed for changing the picking and dropping positions of pot seedlings, handling 20 pot seedlings per min without causing damage to the paper pots. Contrasting with the aforementioned studies, a specialized transplanting manipulator based on steel fingers was designed for hydroponic systems [62]. This system was able to pick up plug-seedlings and place them into culture ducts. The results showed the success of the transplanting operations, achieving 90% of the distribution rate for the leafy vegetable plug-seedlings. Another independent study proposed to design, develop, and test a needle gripper and a two-finger gripper for vegetable transplanting [63]. In short, both gripper types facilitated successful transplanting, but their impact on subsequent plant growth was negligible. However, the study identified several design considerations aimed at enhancing the performance of these grippers for future applications. Most recently, an approach integrated a YOLO-based algorithm into a transplanting robot to assess transplant quality [64]. In field tests, the approach achieved high efficiency and precision of 93% while detecting 180 plants/min.

#### 3.5.7. Fertilizing

Fertilization plays a key role in specialty crop production, as it provides plants with the necessary nutrients for their physiological and morphological development, ensuring optimal productivity. However, a challenge arises in applying the proper fertilizer amount to each plant individually, or even to small plots. Notably, this is even more difficult when using traditional machinery. In addition to fertilization variability, these machines can also cause direct damage to the plants, which would further exacerbate the challenges associated with specialty crop cultivation. A potential solution to this task is the implementation of agricultural robots. These robots can apply fertilizers precisely and efficiently while meeting the specific needs of the plants, especially variable-rate technology (VRT)-based robots. Certainly, equipping these robots with intelligent systems and advanced sensors will facilitate efficient fertilizer application, thereby reducing wastage and environmental impacts traditionally associated with conventional fertilization methods.

Fertilizing robots represent a recent and emerging field in scientific literature, yet they are promising in overcoming current challenges facing specialty crop fertilization. An integrated robotic tool was proposed to detect individual crop deficiencies and respond on a single-crop basis [65]. The method demonstrated efficiency, achieving low localization errors ranging from 4 to 13 mm. This precision enabled the development of robotic fertilizer application with great accuracy, without adversely affecting the plants. Building upon this work, an improved approach developed a platform capable of locating and characterizing single plants along a crop row using point-cloud navigation, LiDAR for crop volume assessment, and multispectral images to evaluate crop health [20]. This approach enabled informed decisions regarding fertilization on a per-plant basis, with liquid fertilizer applied around targeted plants. However, challenges were noted, particularly during early crop stages where plant height differentiation was minimal, and at the end of the season when leaf overlap hindered individual plant separation. Subsequently, a novel method for developing robotic fertilization tasks in crop rows was introduced, leveraging automatic vegetable detection and characterization (DaC) through an algorithm based on artificial vision and a CNN, alongside a low-cost RGB sensor [66]. This approach achieved promising results, with the neural network demonstrating an accuracy of 90.5% and low error percentages (<3%) during vegetable characterization.

### 3.6. Commercial Availability of Robots for Specialty Crops

Robotic advancements represent a significant milestone in modern agriculture, promising greater efficacy, precision, and sustainability in farming applications. Three main factors drive the growing trend in the research on and adoption of agricultural robots: firstly, lower costs compared to heavy machinery; secondly, reduced dependence on human labor; and thirdly, greater precision offered via the equipment. Although there is no precise data on the number of agricultural robots on the market, market projections based on trends, technological advancements, and economic factors indicate that the agricultural robotics market is expected to grow from $5.99 billion in 2022 to $30.5 billion by 2032, reflecting significant expansion in an emerging market [67].

A large number of companies have been involved in the development of agricultural robotics, with approximately 250 companies operating in the “crop robotics” segment in 2022 [68]. Two years later, this number increased significantly, with almost 100 additional companies entering the market [69]. Collectively, companies in this sector raised approximately $400 million in the first half of 2024 [70]. While many of these companies and startups are headquartered in the United States, there is a notable concentration in the Midwest and Pacific regions. These companies have pioneered products tailored to specific crops and different agricultural operations, including autonomous guidance, weed and disease detection, fruit counting, weed spot-spraying, laser weeding, computer vision, and autonomous fruit harvesting, among others. Within this scenario, many companies are notable for their successful fundraising efforts, highlighting the competitive drive of autonomous robotics. For example, Naïo Technologies secured $33 million, SwarmFarm Robotics raised nearly $8.3 million, Burro raised $24 million, and Farm-ng acquired $10 million [69]. Table 1 highlights some of the commercially available robotic solutions, showing a wide range of functionalities designed to meet different agricultural needs. Notably, some of these solutions incorporate renewable energy sources, underlining the industry’s commitment to sustainability. Furthermore, the scalability and adaptability of these technologies are evident, as they cater to a wide range of agricultural operations. Overall, the widespread adoption of robotic solutions in agriculture represents a transformative shift towards more efficient, sustainable, and technology-enabled farming practices.

### 3.7. Case Studies of Agricultural Robot Adoptions for Specialty Crops

In addition to the numerous scientific contributions to agricultural robotics for specialty crops, research centers, and extension programs have also focused on adapting and integrating existing robotic technologies into the world of specialty crops. This approach will undoubtedly bring these technologies closer to the farm, assist in decision-making, and, as a result, encourage wider technology adoption across the industry.

In a recent collaboration, researchers evaluated the operation quality of a spraying robot at a black currant plantation [85]. The robot was equipped with an air-blast spraying system, which allows for application-angle flexibility during the task. They highlighted that, while the oscillating mechanism of the air-blast system offered flexibility during the spraying operation, it contributed to an inaccurate coverage area. Clearly, this strategy does not benefit this type of plantation. A similar approach evaluated the spray patterns and efficiency of a robot in vineyards and apple fields [86]. The researchers found a customized solution based on each crop, for example, a 45° nozzle-heading angle for vineyards and a 90° angle for apple orchards.

Additionally, ongoing projects are also being advanced on integrating spraying robots for specialty crops. For instance, the Institute for Integrative Precision Agriculture (IIPA) at the University of Georgia has been focusing on research in robotics and automation to support specialty crop production [87]. A range of commercial robots has been deployed to assist with tasks such as spraying, mowing, and proximal remote data acquisition. For example, a spraying robot has been evaluated for its performance with both bar and air-blast sprayer systems. Preliminary findings indicated notable effectiveness for both systems; however, their performance varied for different crops. For instance, while the atomizer achieved uniform application over extended distances and smaller crops, such as carrots, the bar sprayer demonstrated significant potential for maintaining vertical uniformity, an important requirement for trellised crops such as cucumbers [88]. Meanwhile, the IIPA is also exploring a commercial robot (Solinftec Solix, São Paulo, Brazil) for its efficiency in spot-spraying and scouting in broccoli fields. Simultaneously, another commercial robot (Farm ng Amiga, CA, USA) is under investigation for its potential to carry sensors to support the proximal monitoring of blueberry fruit development.

Furthermore, researchers have developed a smart-spraying robot system designed to target weeds specifically in raised soil beds and row middles (the areas between rows of plastic-covered beds) [89]. Initially, this system has been applied to fields of peppers and tomatoes. It features an integrated artificial intelligence (AI)-driven algorithm within a camera to identify plants accurately, while an intelligent spraying system targets and treats detected weeds without affecting the vegetable crops. Their results to date demonstrate a 98% accuracy in distinguishing pepper and tomato plants, along with an 85% accuracy in weed detection. The researchers mentioned the system as an improvement over existing spraying methods, making it, therefore, more feasible to adopt.

Moreover, efforts have been made to develop a robotic pollination system for pollinating apples in commercial orchards [90]. The system consists of a camera with AI models to detect flowers and a manipulator arm to spray the pollen solution. Their approach initially achieved 56% success when using R-CNN models. However, after integrating YOLO, they were able to achieve 91% for flower detection and 84% for the pollination rate. The authors emphasized the importance of robotic pollination, stating that it will help avoid flower thinning and minimize the need for fruit thinning in orchards.

Ultimately, a noteworthy project to develop a harvesting robot for blackberries has been advanced [91]. The researchers have focused on developing a vision system to identify berry fruits and also to detect their level of ripeness, which guarantees a selective harvest with estimative yield information for the next round of harvest. Currently, their model achieves 94% accuracy in detecting ripe berries, 91% for ripening, and 88% for unripe berries. Their ongoing project is working on a prototype gripper to grasp and pick berries with high quality, avoiding squeezing and damaging them.

## 4. Discussion

### 4.1. Scientific Contribution of Robots for Specialty Crop Production

Our analysis highlights the contributions of agricultural robots to specialty crops, extending beyond technological advances and arriving at their strengths, challenges, and prospects. The impacts they cause extend further than their efficiency in the field, also encompassing the personal and economic impacts they produce. Harvesting robots have attracted great interest in the scientific community. Their great interest is likely attributed to the labor-intensive nature of the task. In turn, these robots guarantee the health and quality of fruit and vegetables, thus protecting livelihoods and strengthening food security [92,93]. Additionally, they promote the equitable distribution of resources, which will mitigate the risks of food shortages [94]. Therefore, by integrating this type of robot, we will also have a positive impact on the social dynamics of farming communities. Furthermore, this will empower farmers by making them ready and resilient against environmental challenges and economic uncertainties. In essence, merging technological innovation and social sustainability principles will improve agricultural practices, and create communities more prosperous and resilient to promote a more equitable and sustainable future for all [95].

### 4.2. Guidelines and Position Statements

Based on the literature, our review yielded position statements intended to understand the complexities that agricultural robots face during a successful application for specialty crops. These statements include aspects such as sensor deployment, decision support systems (DSSs), and policy considerations. Notably, these complexities are commonly encountered when introducing new technologies into the agricultural context [96]. Consequently, by sharing our insights on the literature, stakeholders will be equipped with valuable information for the effective implementation of technological strategies. Moreover, stakeholders can also find information to establish standards. For instance, guidelines similar to those found in widely recognized frameworks such as the International Organization for Standardization (ISO), will be crucial. These standards can provide a common ground for assessing the reliability, safety, and interoperability of robotic systems in agricultural sceneries. By integrating these guidelines into regulatory frameworks, policymakers can foster an environment that supports innovation while safeguarding both farmers and the environment. Industry leaders, in turn, can utilize these standards to align their technological advancements with sustainable and precise agricultural practices.

### 4.3. Techno-Economic Feasibility

Despite the many models and applications of agricultural robots, there is a major concern about their feasibility. In particular, it relies on the technical and economic impacts of the robot technology. For example, some robots, such as those used as carriers for sprayers, mechanical weeders, and fertilizers, might be easier to deploy immediately due to their broad task performance requiring minimal intervention. However, if they perform site-specific tasks such as spot-spraying, targeted weeding, and plant-level fertilization, they will require even more technological advancements and operational flexibility. The same applies to harvesting, pruning, pollination, and transplanting robots, which are highly dependent on crop patterns. Consequently, it is noteworthy that different robotic applications lead to different impacts on feasibility, justifying the intensity of ongoing research and development (R&D) in these areas. Clearly, robots that perform routine tasks tend to be integrated more quickly and save intensive human labor, whereas non-routine tasks will significantly increase the cost of the robot due to their specificities [97].

Technically, scientific research and field applications have proven that agricultural robots can perform various tasks in the field and maintain the quality of crops, a notable advancement compared to manual and traditional machinery work. Consequently, economic feasibility arises, and many studies have focused on it. For instance, a comparative analysis of the economic feasibility of autonomous field machines compared to conventional machines found a 28% reduction in machine ownership costs and a 34% reduction in machine operating costs [98]. This is due to the lower price of smaller machines, the reduced need for human labor, and the precision in the application of AI-driven equipment. An interesting study reviewed the literature regarding the economic impacts of robots and automation in field crop production [99]. The study highlighted some studies that reported economic analyses of different agricultural robotics. For instance, a particular study demonstrated that a two-row robotic weeder, operating at 80% efficiency, could reduce weeding costs by about 50% compared to manual weeding [100]. The authors concluded that a farmer could invest about 50% more in the robotic weeder and still save costs compared to manual weeding. Similarly, a comparison between conventional weed control with broadcast herbicides and manual inter-row hoeing resulted in cost saving of 12% with the robotic system [101]. Additionally, the authors estimated the investment for a scouting robot and compared it with manual scouting, at the same intensity. The results showed cost savings of about 20% when using the robot. Another study analyzed the profitability of robots for the early seeding and reseeding of sugar beets [102]. The study found that using a robotic seeder, which can plant 4 weeks earlier than conventional equipment, could increase yields by 2.5%. This increase could be as high as 5% if a reseeding task was adopted [102]. However, the authors emphasized that the most feasible system is early seeding because it only includes the cost of seeding once, without additional seeding. With early seeding, it was possible to increase the gross margin by 7.7%, while with reseeding, the corresponding margin was 6.5% [102]. In summary, agricultural robots are economically feasible, but the timeframe for a return on investment depends on the specific application and intensity of use.

### 4.4. Implications and Future Directions to Facilitate the Widespread Adoption of Robots

The exponential growth observed in the adoption of agricultural robots for specialty crops underscores their potential and relevance in solving common problems in these crops. Particularly, merging advanced technologies, computer vision, and AI algorithms has led to the popularization of robots. Additionally, their ability to reduce environmental impacts traditionally caused by heavy machinery, as well as their potential to reduce labor intensiveness, improves their progress. Consequently, if adoption is widespread, it will significantly increase their benefits, along with the quantity and quality of information available, ushering in a new era of autonomous agricultural practices. Henceforth, new research must emerge to further explore these technologies, integrate existing methodologies, and meet the challenges that lie ahead.

#### 4.4.1. Advantages

Based on our review, robotic technologies offer numerous advantages in specialty crop production, particularly in tasks such as harvesting, spraying, pruning, weed control, pollination, transplanting, and fertilizing. One of the primary benefits is precision and efficiency. Agricultural robots equipped with camera systems and AI-driven models can accurately detect and harvest crops, ensuring precise operations that lead to higher quality and yields [36]. By automating tasks traditionally performed through human labor, robots will reduce the dependence on manual labor, consequently saving time and labor costs. This automation also results in constant performance, as robots perform tasks without fatigue or variation. Additionally, automating labor-intensive tasks relieves workers from physically demanding and repetitive work, allowing them to focus on other aspects of crop production and management. This improves the working conditions for agricultural laborers and contributes to overall farm efficiency and productivity. Furthermore, the adaptability of robot technologies is a significant advantage. Robots can be tailored to specific crop types and cultivation practices, making them versatile tools that can address the unique challenges of specialty crop production. For instance, in tasks such as weed control and pruning, robots can be programmed to adjust their operations based on the specific needs of different crops, thereby optimizing resource utilization and minimizing waste [45,48]. Moreover, it is important to understand that robotic technology is not intended for individual operation. Instead, it can be seamlessly integrated with other technologies, such as drones and other autonomous systems. This integration augments the capabilities of agricultural operations by providing a comprehensive solution to various challenges encountered in specialty crop production. For instance, drones equipped with advanced imaging sensors can collect data on crop health and growth patterns. Subsequently, robots can be leveraged to perform tasks such as spraying, pruning, and fertilization. Through the combination of these technologies, farmers can reach efficiency, precision, and efficacy in managing their crops, ultimately resulting in enhanced yields and profitability. Conversely, while autonomous robots are not fully ready for use in certain tasks, efforts have been made to develop collaborative robotics (co-robotics). This eliminates the need for human workers in some tasks, as in the case of controlling harvesting platforms, such as lifts, further improving the overall efficiency of the platform [103]. Although it is not a fully automated task, it has significantly reduced labor. Furthermore, robots sometimes cannot perform a task by themselves. However, they can serve as a platform to transport other equipment, such as drones, within a field to perform a desired task [104].

#### 4.4.2. Limitations

Despite their advantages, agricultural robots in specialty crop production also face several challenges. One significant limitation is the limited application of existing methodologies across different crop types and environmental conditions. Variations in morphology, dimensions, and the growth characteristics of different crops emerge as a recurrent challenge for robotic systems, therefore necessitating customized solutions for each crop. Similarly, irregular terrain and weather variations make their usability even more complex, thus affecting their effectiveness in a practical sense. Resource intensiveness is another challenge; powerful sensors and sophisticated algorithms are constantly being implemented in robotic systems. In turn, deploying such robotic technologies in resource-deficient settings will be a challenge. Additionally, the lack of in-farm experimentation with natural growing fields limits the widespread adoption of these autonomous systems. Clearly, the lack of detailed information on the effectiveness of robotic systems negatively affects the interest in the technologies. Such factors create among farmers a resistance to incorporating the growing range of robotic solutions for specialty crop production within their future investment plans, considering the great expectations they have. Otherwise, this research strategy would provide stakeholders with complete information on the robot’s performance and reliability over such a large area as a typical farm field. Technical challenges, such as speed in decision-making and compatibility with current agricultural machines and infrastructure, can also impact robotic systems performance and increase scaling challenges. Furthermore, the geographical spread of such robotic technologies in specialty crop fields is limited. Among the most significant reasons for this adoption gap is the high price involved in acquiring such technologies. Concurrently, it presents a potential opportunity for international and inter-institutional collaboration.

#### 4.4.3. Future Research

To address the challenges facing agricultural robots in specialty crop production, future R&D should focus on several key areas. Some of these areas are highlighted below:

- **Developing multi-purpose robotic systems:** Such robots could be capable of performing a variety of tasks in the field. For example, a previous study integrated a robot for harvesting and pruning strawberries [105]. Certainly, these types of robots will also perform these tasks with different types of crops, growth patterns, and environmental conditions. As a result, they will perform more reliable tasks in real-world settings.
- **Swarm robotics:** It is important to recognize that just one robot may not be enough to perform a task instantly; instead, the concept of a swarm of robots working together should be adopted. This approach can enhance efficiency, resilience, and versatility in tackling the challenges faced in specialty crop production, even in complex and dynamic agricultural environments.
- **Advances in vision systems:** Advances in vision systems, including multispectral and hyperspectral cameras, have increased significantly. Simultaneously, efforts have been made to analyze these large datasets. For instance, the application of CNN and YOLO are noteworthy architectures for identifying patterns in images. However, opportunities can emerge by applying the spatial, spectral, and texture-aware attention network (SSTNet) [106] and the dual-branch collaborative learning network (DBCLNet) [107]. These models contribute directly to classification, segmentation, temporal data analysis, and the identification of complex patterns.
- **User-friendly tools:** User-friendly tools aim to enhance the accessibility and usability of robotic systems for farmers and agricultural workers. Consequently, a collaboration between researchers, extension programs, industry stakeholders, and agricultural practitioners is essential to accelerate the adoption of the technology into agricultural practices.
- **Power systems:** For the most part, the electric system is predominant. This is undoubtedly an advance on common fuel systems. Consequently, their productivity can be enhanced with efforts made with charging stations. Furthermore, greater investment can be also made in solar power systems, for example [80,84]. This type of power supply both contributes to sustainability and increases the operability of robots.
- **Connectivity and data exchange:** As robotics are rapidly emerging, it is also important to consider the connectivity between them and existing agricultural machinery. This includes establishing connectivity and data exchange protocols to support their coordination and data value [108].
- **Artificial intelligence models:** Integrating ML and DL models has enhanced the capabilities of agricultural robots to perform tasks. Implementing further technologies such as the Internet of Things (IoT) and large language models (LLM) can facilitate real-time problem-solving, moving toward agriculture 5.0 [109,110]. Additionally, these emerging technologies will improve user adoption and understanding [111].
- **Priority subjects for further investigation:** Research on agricultural robots will be intensified in areas such as harvesting, pruning, pollination, and transplanting. Harvesting tends to continue with a prominent focus. Pruning and pollination might be prioritized due to the high impact on yield and quality. Subsequently, investigations into transplanting will be intensified to reduce this labor-intensive task.

### 4.5. Conclusions

As highlighted in this study, the technological level of agricultural robots is already sufficiently advanced today. However, it needs to be introduced into a concept of applicability combined with sustainability to guarantee effectiveness for specialty crop production. If our perspectives hold true, the direction of agricultural advancement will require the efforts of policymakers, researchers, and industries. Consequently, as agricultural robotics progresses, its benefits will involve not only productivity but also the transformation of the agricultural sector as a whole, making it more sustainable, equitable, and resilient for the next generation.

Finally, we provide responses to the initial questions:

**i.**  **What is the current state of research on agricultural robots tailored to specialty crops in the literature?**
  Research on agricultural robots for specialty crops has grown substantially in the past decade, with scholarly contributions increasing sixfold.
**ii.**  **Which journals are most active in publishing research on agricultural robots for specialty crops?**
  The most active journals include *Computers and Electronics in Agriculture* (Elsevier), *Agronomy* (MDPI), *Biosystems Engineering* (Elsevier), *Sensors* (MDPI), and *Agriculture* (MDPI), collectively comprising almost 40% of the relevant literature.
**iii.**  **Which countries are the primary contributors to scientific publications on agricultural robots for specialty crops?**
  Primary contributors to scientific publications on agricultural robots for specialty crops extend primarily to China, followed by the United States, and Japan.
**iv.**  **Which specific crops derive the most benefits from agricultural robots?**
  Apple and tomato crops are among the most promising crops for applications of agricultural robots.
**v.**  **What are the primary tasks for which agricultural robots are increasingly being deployed in specialty crop production?**
  Agricultural robots primarily target harvesting, also with growing attention to spraying, pruning, weed control, pollination, transplanting, and fertilizing tasks in specialty crop production.
**vi.**  **What are the benefits, limitations, and future research opportunities in the field of agricultural robots for specialty crops?**
  The benefits of agricultural robots in specialty crop production include precision, efficiency, reduced labor costs, and improved crop quality. Limitations include crop-specific challenges, environmental dependency, and high acquisition costs. Future research should focus on adaptability, field testing, resource efficiency, and integration with existing infrastructure.

## Figures and Tables

**Table 1:** Companies innovating in the autonomous agricultural robotics markets. Robots are displayed in alphabetical order by company.

Company	Product	Description	Reference
>_farm-ng	*Amiga* 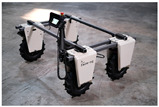	Autonomous modular platform robot. Can be used as a non-autonomous remotely controlled. Carries up to 450 kg and has 6–7 h of runtime on heavy payload.	[71]
Agrobot	E-Series 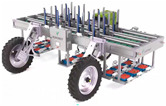	Autonomous harvesting robot. Can use up to 24 independent robotic arms working simultaneously. Applied specifically for strawberry harvesting.	[72]
Burro	*Burro* 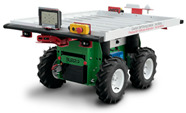	Autonomous payload robot. Used for carrying payload of up to 226 kg and towing payload of up to 453 kg.	[73]
GUSS automation	*GUSS* 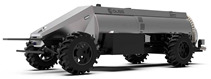	Autonomous diesel-powered sprayer. Suitable for use in orchards, vineyard, and high-density orchard. Carries a spray tank with a capacity of over 2000 L. Running time of about 13–14 h.	[74]
Harvest CROO	*Robot* 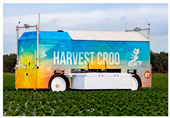	Autonomous harvesting robot. Applied specifically for strawberry harvesting.	[75]
MQ Autonomous Agritech	*M200* 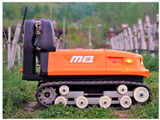	Autonomous spraying system. A compact, electric solution tailored for orchards and vineyards. Carries a 200-L tank. Supports 200 kg of cargo and 500 kg of towing. Autonomy 3–6 h of runtime.	[76]
Naïo Technologies	*Orio* 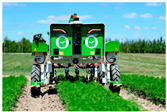	Autonomous multi-platform robot. Carries up to 1450 kg of tools in the toolbar. Can achieve production of up to 6 h/day.	[77]
Odd.Bot	*Maverik* 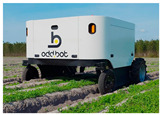	Autonomous mechanical in-row weeding. Intended to be applied mostly in organic crops. Up to 2 weeds per second per arm.	[78]
Robotics Plus	*Prospr* 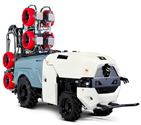	Autonomous spraying robot. Developed for fungicide and herbicide applications. Minimum row spacing is 1.83 m.	[79]
Solinftec	*Solix* 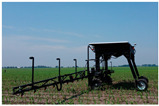	Autonomous weed detection, data collection, and spot-spraying robot. Solar powered. Runs using solar energy during daytime and batteries during nighttime.	[80]
Swarm Farm	*SwarmBot 5* 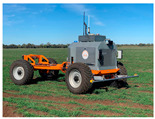	Autonomous multi-task robot. Can be used for any agricultural task including spraying, mower, slasher, spreader, plating, harvesting, and cultivation.	[81]
Tortuga Agtech	*Tortuga* 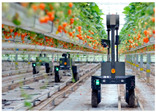	Autonomous harvesting robot for strawberry or table grape. Can also be used for UV-C treatment and trim plants.	[82]
Twisted Fields	*Acorn* 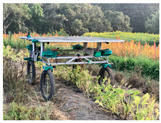	Autonomous open-source robot. It is solar-powered and equipped with a three point hitch for implements. Can operate in tasks such as soil preparation, planting, weeding, harvest aid.	[83]
XAG	*XAG R150* 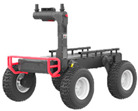	Autonomous multi-purpose robot. Can be used as a sprayer, air blast, carrier, and mower.	[84]

## Data Availability

The dataset can be found at Mendeley data: https://doi.org/10.17632/482j99rfpz.4.

## Abbreviations

3D	tree-dimensional
AI	artificial intelligence
CNN	convolutional neural network
Co-robotics	collaborative robotics
DaC	detection and characterization
DBCLNet	dual-branch collaborative learning network
DL	deep learning
DoD	drop-on-demand
DSS	decision support systems
FWCI	field-weighted citation impact
IIPA	Institute for Integrative Precision Agriculture
ISO	International Organization for Standardization
IoT	Internet of Things
IWM	integrated weed management
LiDAR	light detection and ranging
LLMs	large language models
ML	machine learning
MOTS	multi-object tracking and segmentation
PRISMA	Preferred Reporting Items for Systematic Reviews and Meta-Analysis
PVS	precision variable-rate spraying
R&D	research and development
SEPs	stem emerging points
SSTNet	spatial, spectral, and texture-aware attention network
UGVs	unmanned ground vehicles
VRT	variable-rate technology
YOLO	you only look once
